# Clinicopathology of non-infectious choroiditis: evolution of its appraisal during the last 2–3 decades from “white dot syndromes” to precise classification

**DOI:** 10.1186/s12348-021-00274-y

**Published:** 2021-11-17

**Authors:** Carl P. Herbort, Piergiorgio Neri, Ioannis Papasavvas

**Affiliations:** 1 Retinal and Inflammatory Eye Diseases, Centre for Ophthalmic Specialized Care (COS), Rue Charles-Monnard 6, CH-1003 Lausanne, Switzerland; 2 The Eye Institute, Cleveland Clinic Abu Dhabi, Abu Dhabi, United Arab Emirates; 3 Cleveland Lerner College of Medicine, Case Western University, Cleveland, OH USA; 4 Khalifa University, Abu Dhabi, United Arab Emirates

**Keywords:** Fluorescein angiography (FA), Indocyanine green angiography (ICGA), Spectral domain optical coherence tomography (SD-OCT), Enhanced depth imaging optical coherence tomography (EDI-OCT), Multiple evanescent white dot syndrome (MEWDS), Acute posterior multifocal Placoid pigment Epitheliopathy (APMPPE), Idiopathic multifocal choroiditis (MFC), Serpiginous choroiditis (SC), HLA-A29 birdshot Retinochoroiditis (BRC), Vogt-Koyanagi-Harada disease (VKH), Sympathetic Ophthalmia (SO) and sarcoidosis chorioretinitis (SARC)

## Abstract

Choroidal imaging investigation techniques were very limited until 2–3 decades ago.

Fluorescein angiography (FA) was not suited for the analysis of the choroidal compartment and B-scan ultrasonography did not provide enough accuracy. It was on this background that a purely phenomenological approach was attempted to classify these choroiditis diseases by regrouping them under the vague potpourri term of “white dot syndromes”. With the availability of precise investigational modalities of choroidal inflammation or choroiditis-induced lesions, such as indocyanine green angiography (ICGA), spectral domain optical coherence tomography (SD-OCT) and enhanced depth imaging optical coherence tomography (EDI-OCT) it became possible to better classify these diseases based on clinico-pathological mechanisms rather than on purely phenomenological observation.

Recently OCT-angiography has implemented the armamentarium of diagnostic techniques possibly also contributing to the classification of choroidal inflammatory diseases.

Based on pioneering pragmatism, the aim of this article was to give a clear classification of non-infectious choroiditis. Thanks to new imaging investigations of the choroid, it is now possible to classify and understand the diverse clinicopathological mechanisms in the group of non-infectious choroiditis entities.

## Introduction

The choroid has been poorly accessible to imaging investigation until 2–3 decades ago. Indeed, exploration of the choroidal compartment for inflammatory conditions used to be limited before appropriate technologies became available. B-scan ultrasonography was the routinely used method, however, lacking the needed precision for detailed analysis and giving only morphological information without haemodynamic details, being no more cited today as an indication to analyse choroiditis [1]. On the other hand, fluorescein angiography (FA) the classical imaging modality for posterior ocular inflammation, is mainly giving information on the surface of the fundus (retina and optic disc) but cannot provide useful information of the choroid, being ill-adapted for choroidal investigation, since the retinal pigment epithelium (RPE) is a screen to the visible spectrum of light used for FA [2].

Without having the tools to investigate the choroid and simply based on the visual, phenomenological aspect of fundus lesions, a “perspective article” entitled “Fundal white dots: the spectrum of a similar pathological process” was published in 1995 [3].

This article brought together disease groups which, we know now, have nothing in common. The thesis of a “similar pathological process” for such diverse entities as sympathetic ophthalmia (SO), acute posterior multifocal placoid pigment epitheliopathy (APMPPE) or diffuse unilateral subacute neuroretinitis (DUSN) was a pure conjecture with no objective consistency. However, the term of “white dot syndromes” was born and applied to a multitude of conditions allegedly having a similar physiopathology with diverse expressions [3]. The terminology was generated at a time when most of these diseases were poorly known and ill-understood. To be able to include them into one group, was very comfortable for the clinicians and the term was quickly adopted and widely used.

With the availability of more precise imaging methods of the choroid such as indocyanine green angiography (ICGA) and, later, optical coherence tomography (OCT), enhanced depth OCT (EDI-OCT) and OCT angiography (OCT-A), appraisal of choroidal inflammation acquired a crucial gain in accuracy. These methods allowed to precisely determine which structures were involved in the different non-infectious choroiditis entities and made it possible to classify this group of diseases not according to purely phenomenological criteria but according to precise clinico-pathological criteria. Using ICGA, the choroidal structures could be imaged in a dynamic way as the near-infrared spectrum of rays used went beyond the RPE screen allowing to follow the evolution of choroidal ICG fluorescence [4].

ICGA was at the origin of a determining progress especially in choroidal inflammatory diseases [5–7]. A detailed report on the classification and characteristics of non-infectious choroiditis has been published recently [8] and the present introductory article of the special issue of the “Journal of Ocular Inflammation and Infection” is intended to set the stage for the different contributions of this issue on the topic.

## Brief summary on imaging methods exploring the choroid and choroiditis induced outer retinal lesions

### Indocyanine green angiography and ICGA derived classification of non-infectious choroiditis

Indocyanine green angiography (ICGA) relies on two essential characteristics of the ICG molecule used in this type of angiography. ICG fluoresces in the range of 830 nm (nm) in the near infrared spectrum of light radiation (1) and has to be considered as a macromolecule (2) [2].

#### Fluorescence characteristics of the ICG molecule

**Fig. 1:**
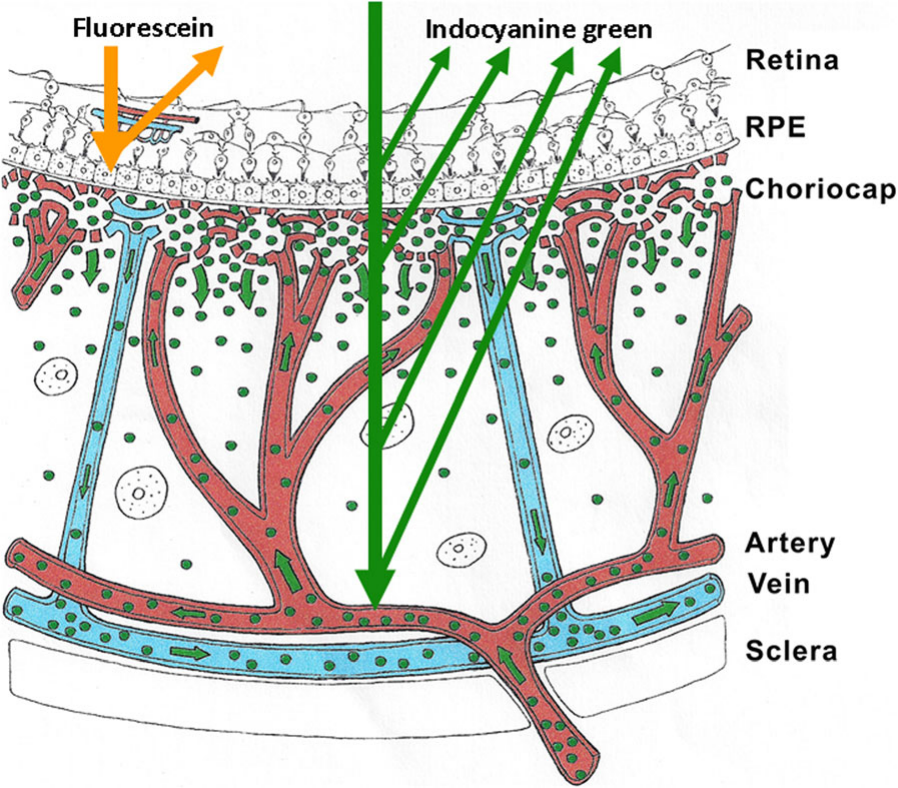
Cartoon on optical characteristics explaining the differences between fluorescein (FA) and indocyanine green angiography (ICGA). FA is only able to analyse the fluorescence coming from the retina as the RPE is blocking visible light fluorescence, while ICGA is able to analyse retinal and choroidal near-infrared fluorescence which is not blocked by the RPE. Note, at the level of the choriocapillaris, the schematic drawing shows the physiological egression of the ICG macromolecular complex from the fenestrated choriocapillaris impregnating the choroidal stroma (see Fig. 2), determining the intermediate and late angiographic phases that are important for the evaluation of choroiditis

Maximum absorption of the ICG molecule occurs at around 800 nm followed by fluorescence emission at around 830 nm which can be detected through the RPE by infrared cameras. In contrast, fluorescein sodium (FNa) fluoresces in the visible light range and its choroidal fluorescence is stopped by the RPE and cannot be detected, making FA inappropriate to analyse the choroid (Fig. 1).

ICG fluorescence is sometimes falsely called *cyanescence* which is an inappropriate and even confusing term, as the basic optical mechanism for both molecules is fluorescence.

#### Macromolecular behaviour of the ICG molecule

The ICG molecule is not much bigger than FNa molecule (775 Da (d) versus 332 d) However the ICG molecule is bound up to 98% to blood proteins reaching a molecular weight of 60′000 to 80′000 d. This large molecular complex does not egress from inflamed retinal vessels nor from large choroidal vessels because of the tight junctions that make these vessels impermeable to large molecules. However, it physiologically egresses from the fenestrated choriocapillaris and impregnates the choroidal space determining the ICGA patterns.

#### ICGA angiographic phases

**Fig. 2:**
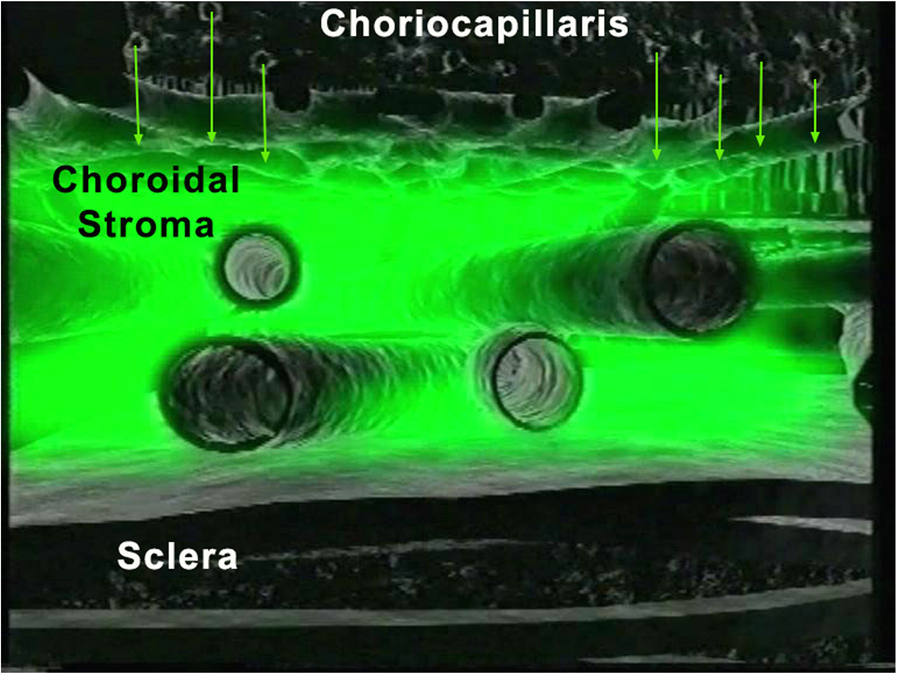
ICGA. Schematic cartoon showing the progressive impregnation of the choroidal stroma by the ICG-macromolecular complex egressing from the choriocapillaris (green arrows)

In the early phase of angiography, ICG fluorescence is detected in the retinal and large choroidal vessels. The macromolecular behaviour of ICG will determine the subsequent phases of the ICGA protocol. Escaping through the fenestrations of the choriocapillaris the large molecular complex will shed into the choroidal space and remain trapped as wash-out will be slow because of the size of the molecular complex (Figs. 1 & 2). Consequently, analysis of ICGA fluorescence is more focussed on the choriocapillaris flow and the subsequent impregnation of the choroidal space by the ICG molecular complex than the intravascular fluorescence. It is by analysing the pattern of impregnation of the choroidal space and its disturbance by choriocapillaris non-perfusion or impairment of ICG fluorescence impregnation caused by inflammatory space-occupying lesions in the choroidal stroma that ICGA made it possible to classify choroiditis and its different mechanisms.

Classical analysis of ICGA comprises early frames showing the circulation in retinal and larger choroidal vessels, followed by the intermediate angiographic phase (8–10′) and the late angiographic phase (28–32′).

#### ICGA angiographic patterns and classification of non-infectious choroiditis

**Fig. 3:**
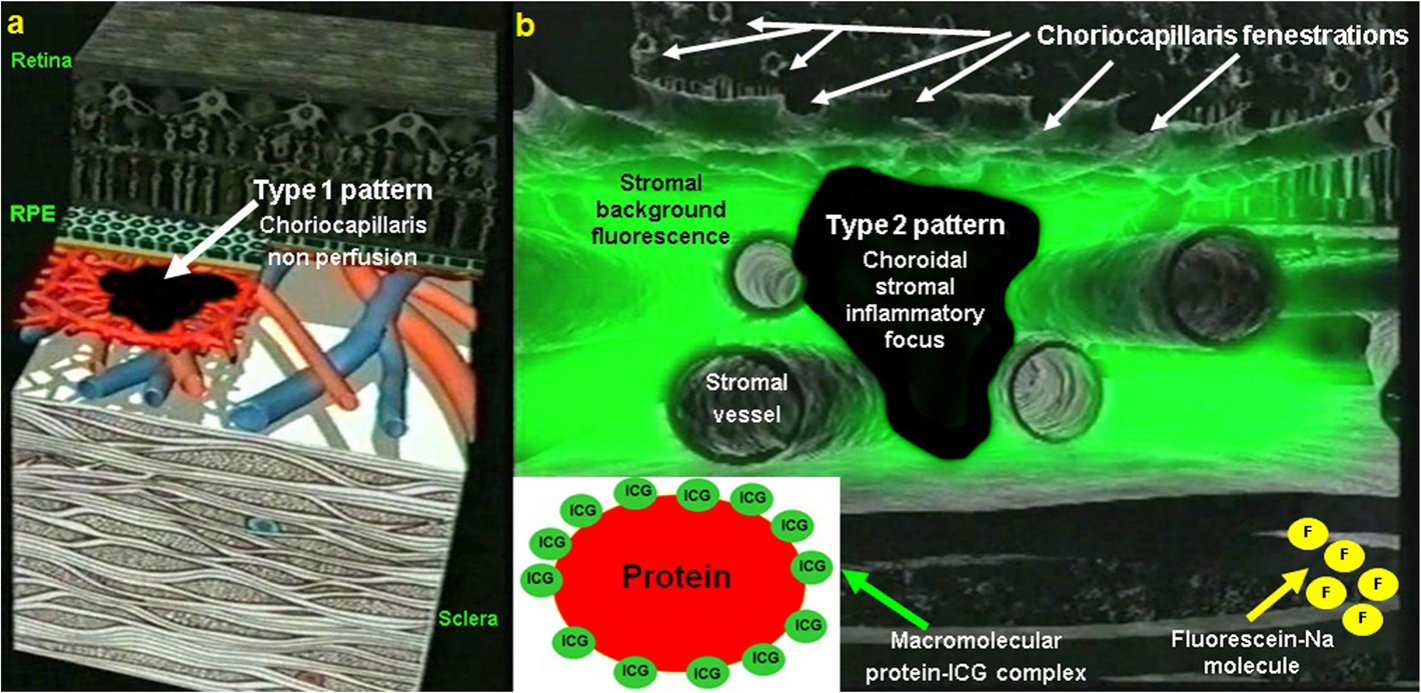
Indocyanine green angiography (ICGA) patterns in non-infectious choroiditis. Two main patterns have been identified in choroiditis. Pattern 1 is generated in inflammatory choriocapillaris non-perfusion (a). Pattern 2 is found in stromal inflammation when foci develop and impair the diffusion of the ICG-protein complex shedding from the fenestrated choriocapillaris (b). The ICG-protein complex is shown in the insert, which is much larger than the fluorescein molecule with a slow wash-out from the choroid. (Reprinted from reference 8)

Two main patterns of ICGA signs in choroiditis have been identified as shown on Fig. 3.

##### Choriocapillaritis or inflammatory choriocapillaropathies

**Fig. 4:**
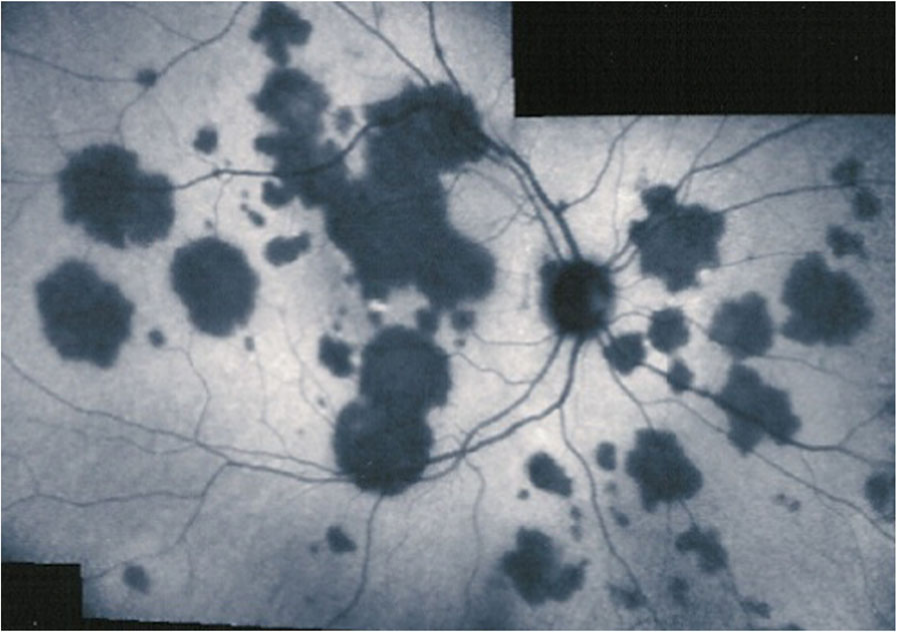
Indocyanine green angiography (ICGA), type 1 pattern: choriocapillaris non-perfusion. Typical geographic areas of dark non fluorescence in case of acute posterior multifocal placoid pigment epitheliopathy (APMPPE) or acute multifocal ischaemic choriocapillaritis (AMIC)

Inflammatory choriocapillaris non-perfusion or hypoperfusion, represented on the left cartoon of Fig. 3, appears as irregular geographic areas of hypofluorescence or absence of fluorescence (Fig. 4). This pattern is found in **primary or secondary choriocapillaritis entities or Inflammatory choriocapillaropathies.**

**Fig. 5:**
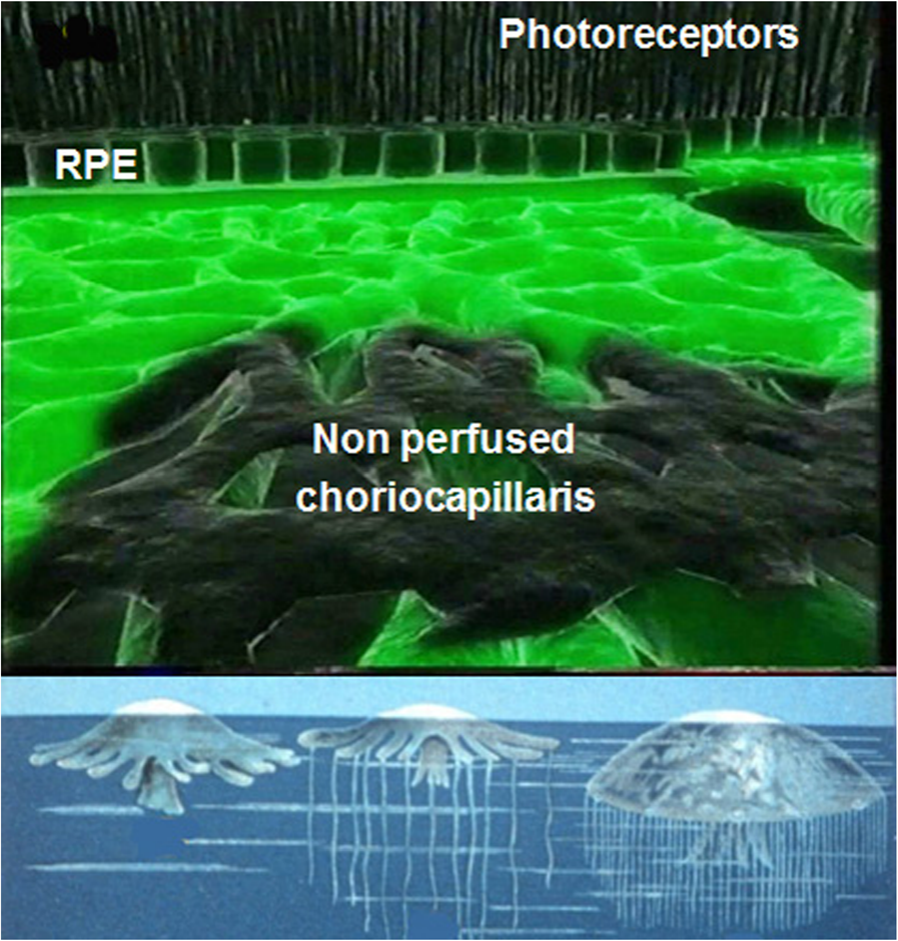
The jellyfish analogy for choriocapillaritis. The lesion process (non-perfused choriocapillaris – top picture) lies immediately under the RPE in the same manner as jellyfishes are lingering under the surface of the water (lower picture)

The ICGA pattern found in choriocapillaritis entities can be represented by the analogy of the “jellyfish constellation” because choriocapillaris non-perfusion, as jellyfishes, linger undetected under the RPE, unless ICGA is performed (Fig. 5). The list of the main choriocapillaritis entities is shown on Table 1.

**Table 1:**
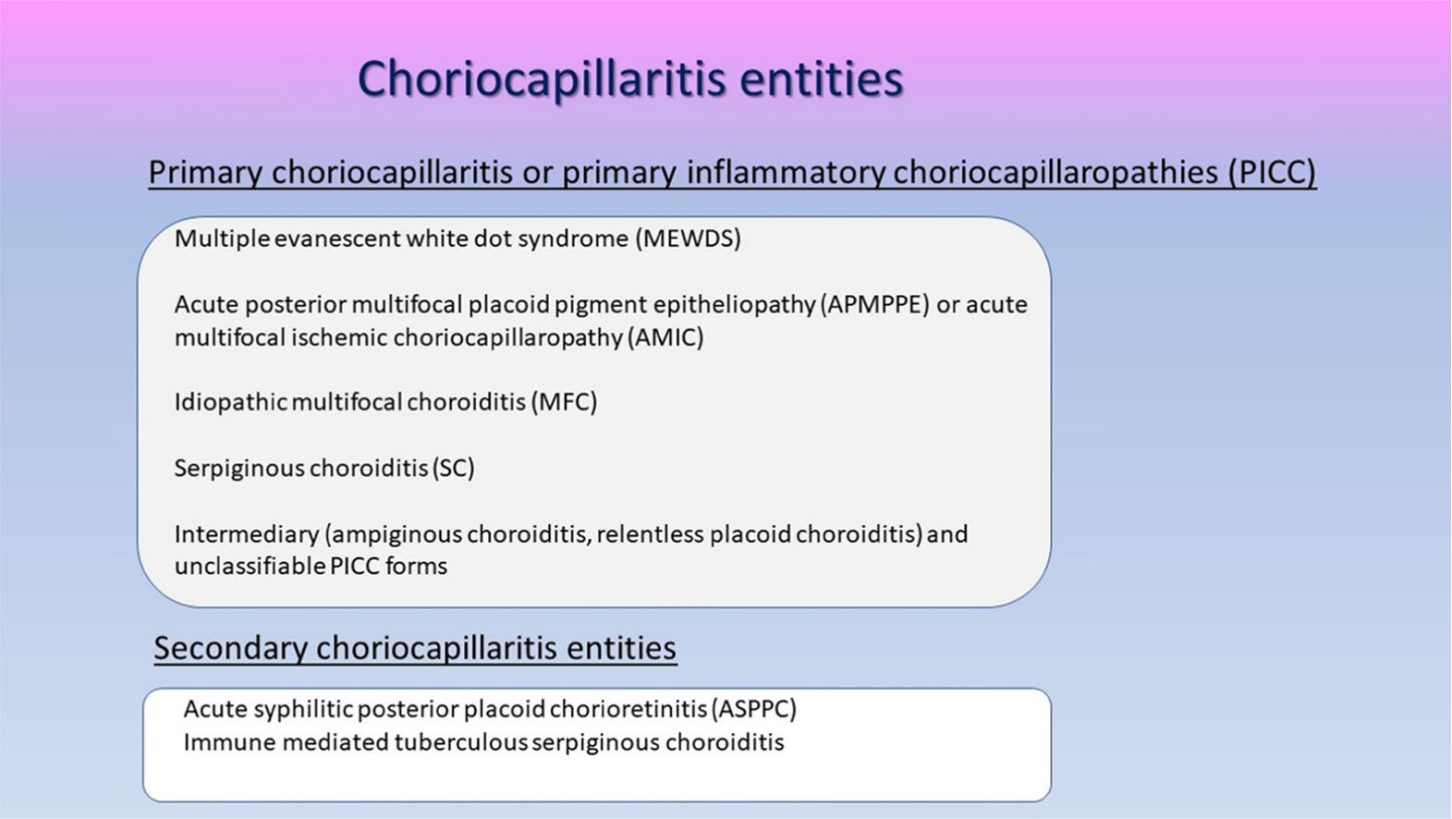
Choriocapillaritis entities

##### Stromal choroiditis

**Fig. 6:**
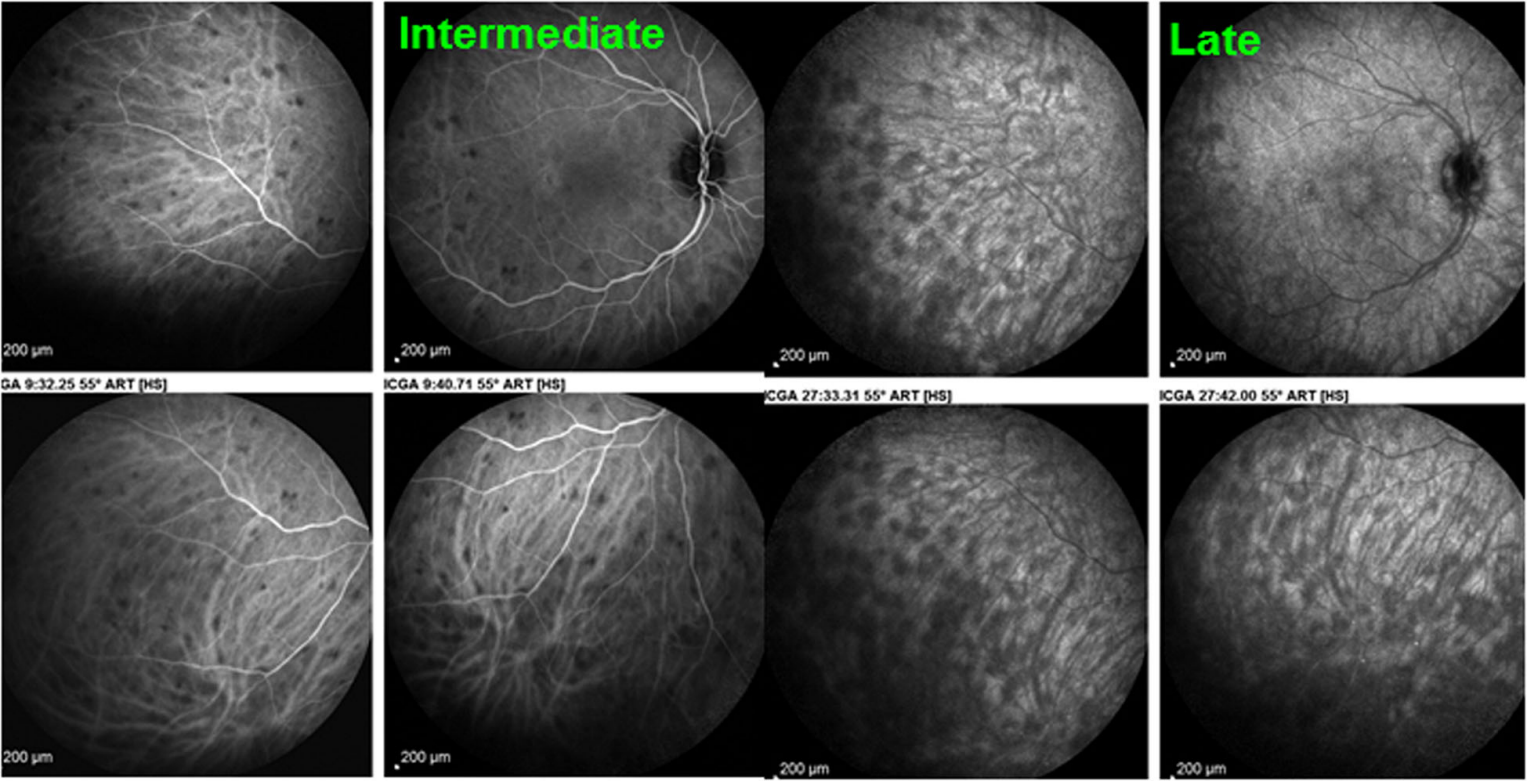
Indocyanine green angiography (ICGA), type 2 pattern found in stromal choroiditis. Typical round HDDs of similar size evenly distributed caused by the presence of inflammatory foci that impair the diffusion of the ICG dye showing granulomas in negative in a case of Vogt-Koyanagi-Harada disease (VKH). HDDs are more clearly delineated in the late angiographic phase (right quartet) than in the intermediate phase (left quartet)

**Fig. 7:**
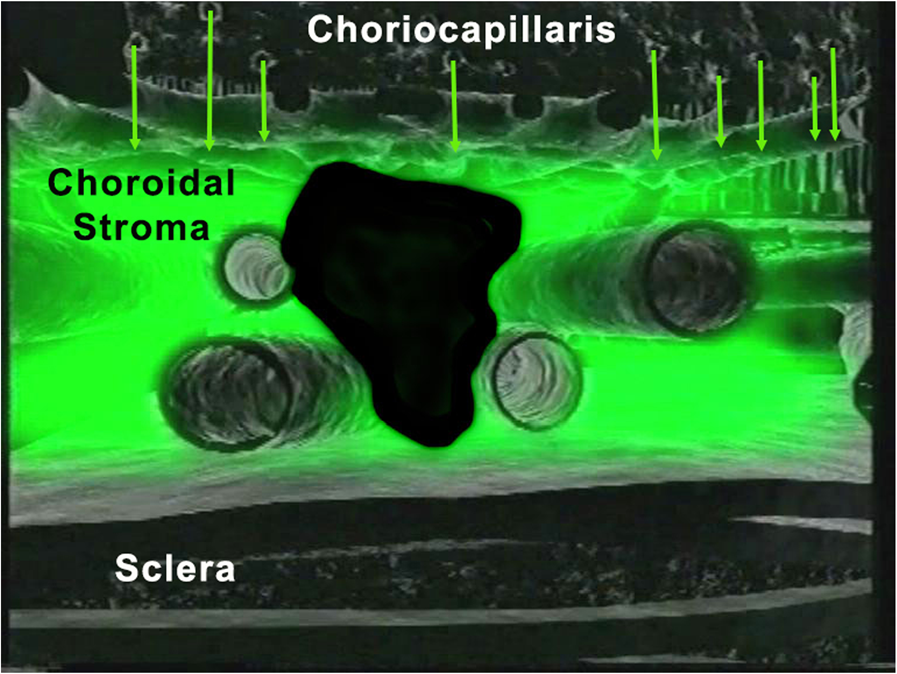
Indocyanine green angiography (ICGA), Cartoon of type 2 pattern found in stromal choroiditis, featuring the presence of a focus in black

On the other hand, **stromal choroiditis** produces type 2 ICGA pattern characterised by round, often numerous and evenly distributed hypofluorescent dark dots (HDDs) **(**Fig. 3**, right cartoon &** Fig. 6**)** generated by stromal inflammatory foci that impair the diffusion of the ICG-protein complex, as shown on Fig. 7.

**Fig. 8:**
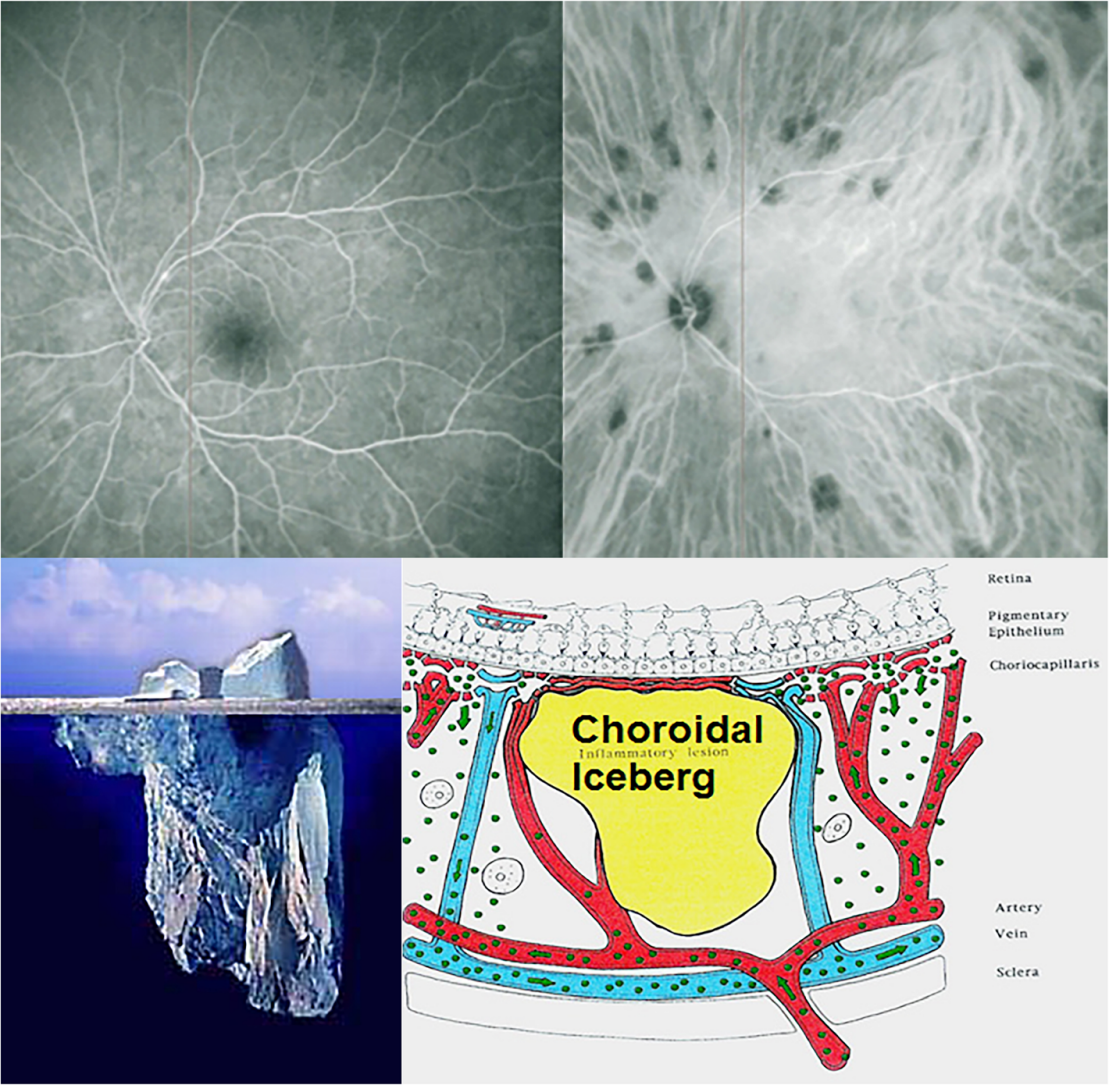
Indocyanine green angiography (ICGA), type 2 pattern: the iceberg analogy for stromal choroiditis. The bottom two pictures show the analogy between the stromal focus and iceberg. The lesion is not seen on FA (top left), but clearly identified on ICGA appearing hidden below the RPE, appearing as HDDs on ICGA (top right)

The analogic comparison for stromal choroiditis lesions is well chosen by the “iceberg constellation”, as choroidal lesions are silent on FA and their extent is only determined by ICGA signs like icebergs barely seen on the surface and for which a major part of the volume is hidden under the water (Fig. 8).

**Fig. 9:**
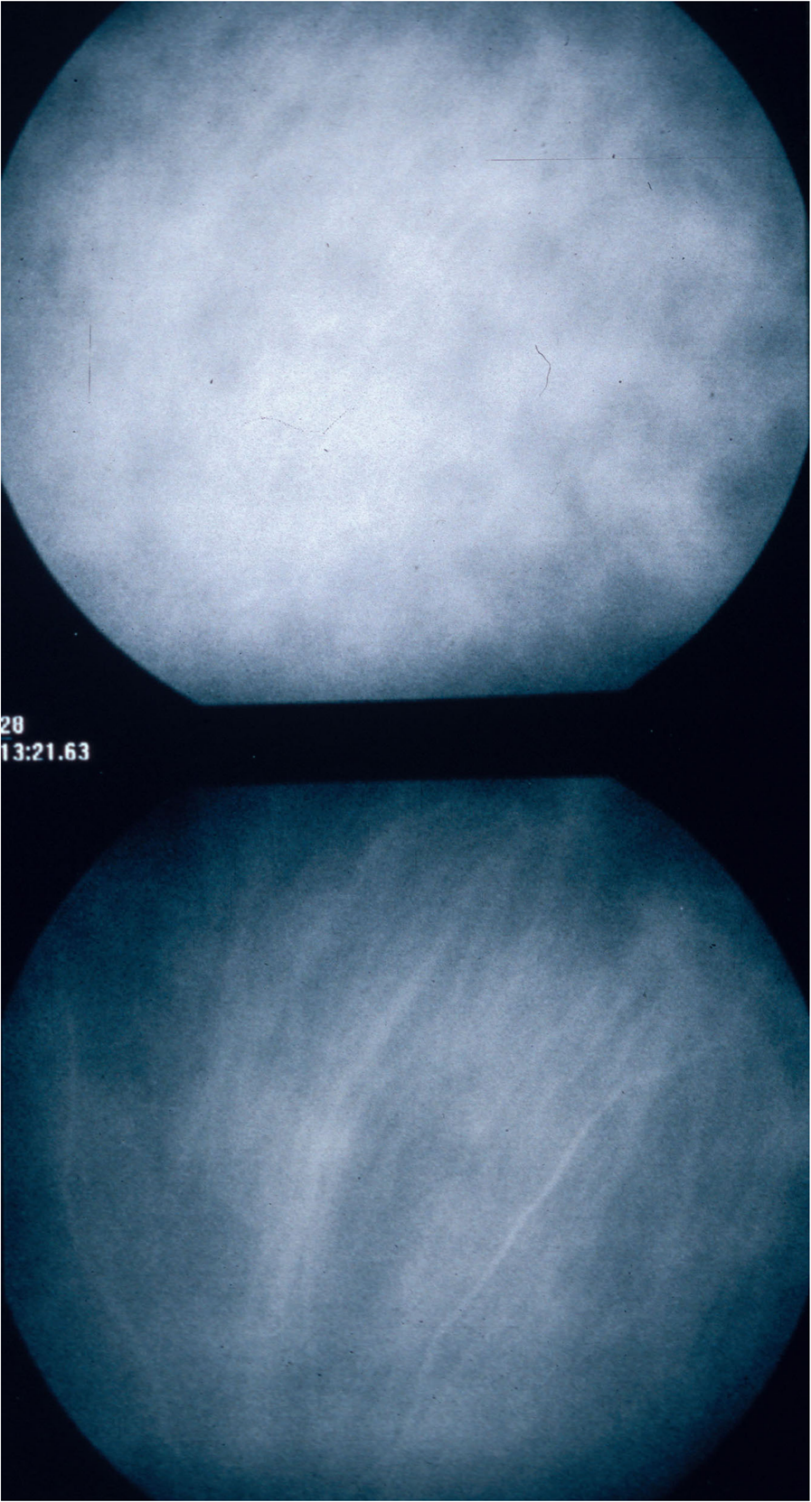
Indocyanine green angiography (ICGA), type 2 pattern found in stromal choroiditis. Typical round HDDs of similar size evenly distributed caused by the presence of inflammatory foci that impair the diffusion of the ICG dye showing granulomas in negative in case of Vogt-Koyanagi-Harada (VKH) disease. Choroidal vessels are no more distinct and appear fuzzy with diffuse hyperfluorescence hiding the HDDs (Top picture). After 3 days of intravenous corticosteroids the choroidal vessels are again distinctly visible, and HDDs have partially resolved

Apart from HDDs, ICGA shows additional leakage from larger choroidal vessels that are less distinct and appear fuzzy (Fig. 9). The whole set of angiographic signs that can be identified in stromal choroiditis is listed on Table 2. A grading of these signs allowed to establish a precise quantitative angiographic score [9]. The main stromal choroiditis entities are listed on Table 3.

**Table 2:** ICG angiographic signs in stromal choroiditis

**1. Hypofluorescent dark dots (HDDs)**	
**2. Indistinct choroidal vessel (Fuzziness of choroidal vessels)**	
**3. Diffuse late choroidal hyperfluorescence (partially hiding HDDs)**	
**4. ICGA disc hyperfluorescence (in severe choroiditis)**

**Table 3:**
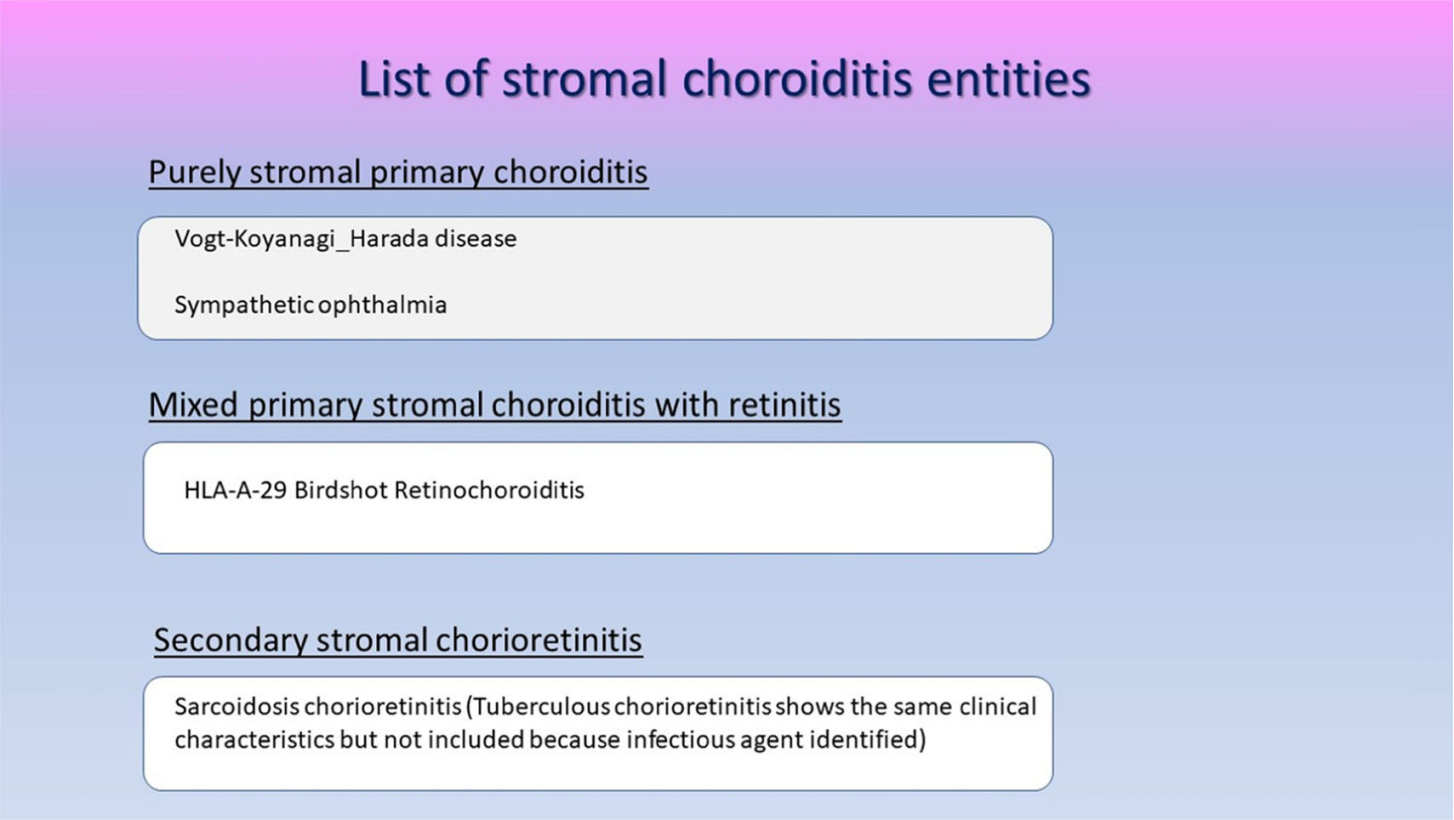
List of stromal choroiditis entities

Unfortunately, all choroiditis conditions were erroneously grouped indistinctly under the general term of “white dot syndromes” in the past, albeit the fact that disease mechanisms were and are obviously different. Such a terminology is therefore inappropriate and obsolete. Consequently, these diseases should be classified into choriocapillaritis (sometimes also called inflammatory choriocapillaropathies) on one side and stromal choroiditis on the other side according to the disease mechanism uncovered by ICGA [10–13].

### Relevant points in other imaging modalities for choroiditis and choroiditis related lesions

#### Fluorescein angiography (FA)

**Fig. 10:**
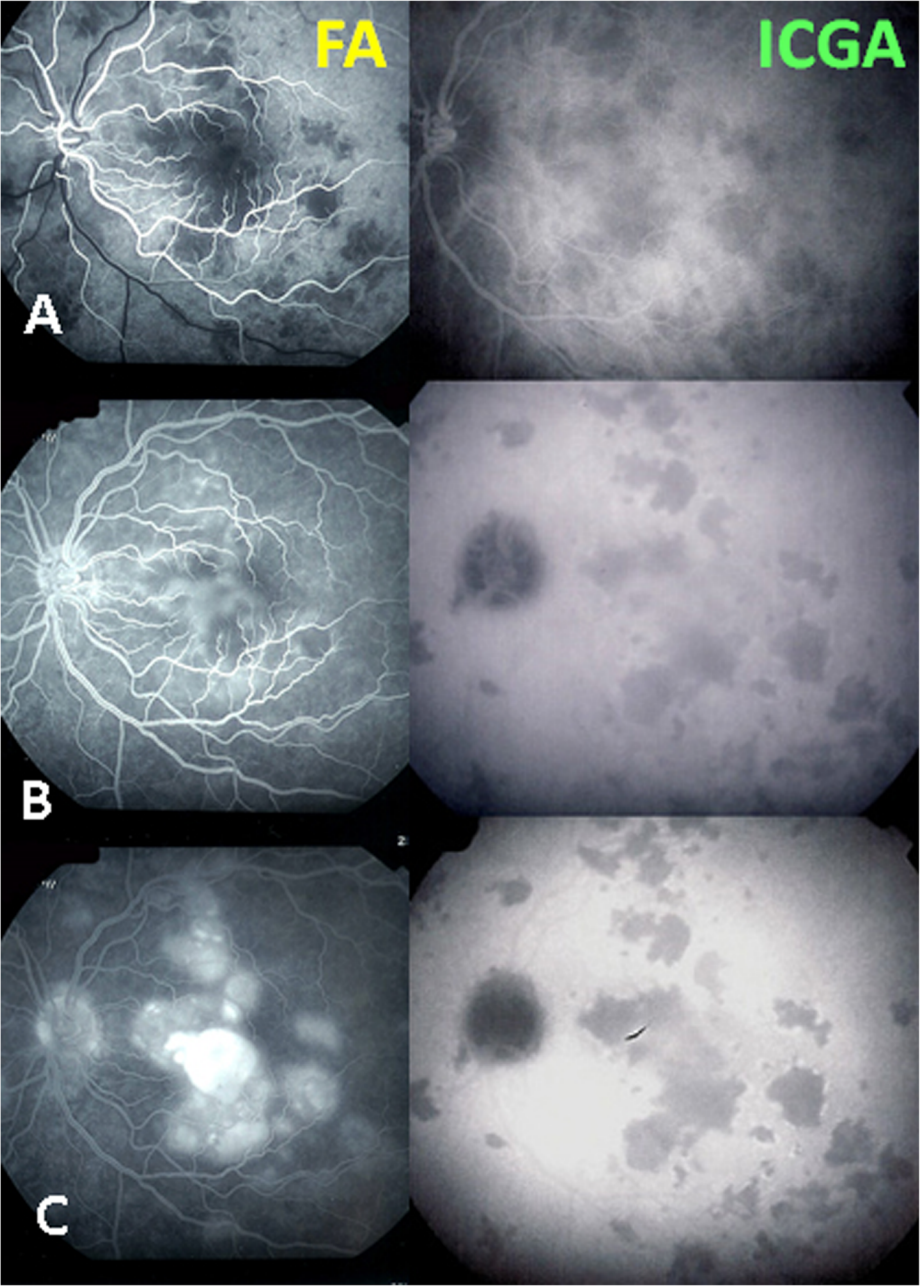
Parallel FA/ICGA analysis of APMPPE, retinal pooling caused by choriocapillaris non-perfusion induced retinal ischaemia. (A) early phases of FA and ICGA both showing non-perfusion. (B) Intermediate phases of FA and ICGA showing persistent non-perfusion in ICGA (right frame) and beginning of pooling in FA (left frame). (C) late phases of FA and ICGA still showing non-perfusion in ICGA (right frame) and massive pooling in FA (left frame)

FA does not play a primary role for choroiditis. In the early angiographic phase, when the injected bolus of fluorescein is highly concentrated it shows the choriocapillaris in the first 40 to 60 s of angiography despite the RPE screen. In this angiographic phase, it can also show choriocapillaris perfusion delay or non-perfusion [14]. In case of severe choriocapillaritis-induced ischaemia, such as in APMPPE, it also shows late retinal pooling that can only come from retinal vessels responding by vasodilatation and exudation, similar to what is happening in diabetic retinal ischaemia. This was substantiated by retinal SD-OCT in a recent report [15]. Indeed, this fluid cannot come from the choroidal space by a so-called change of polarity of the RPE, as has been hypothesised, because there is no fluid in the underlying non-perfused choriocapillaris (Fig. 10) [14]. FA can also show disc hyperfluorescence, a spill-over of the occult choroidal inflammation. However, we have to consider that FA is mandatory in all cases of posterior uveitis as, often, biomicroscopy does not allow to appreciate in detail neither blood-ocular (−retinal) barrier disruption nor retinal vasculitis. Their correct appraisal can be crucial both for the diagnosis and for treatment strategy. This is the case for HLA-A29 birdshot retinochoroiditis where FA is essential to follow retinal disease, as there is dual independent retinal and choroidal involvement.

#### Spectral domain optical coherence tomography (SD-OCT) [swept source OCT (SS-OCT)]

**Fig. 11:**
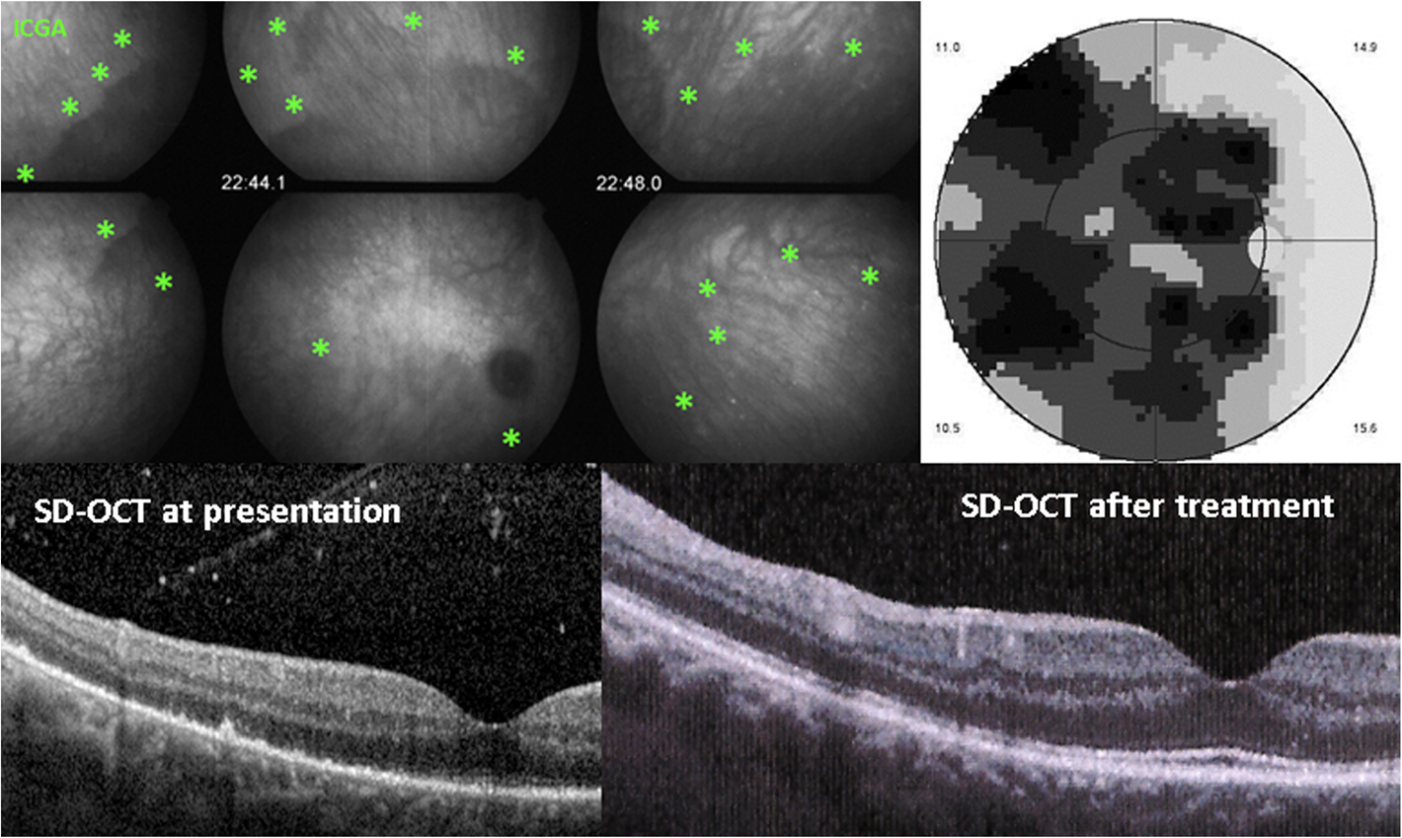
SD-OCT imaging of loss of photoreceptor outer segments in a case of syphilitic secondary choriocapillaritis. Choriocapillaris non-perfusion shown by green asterisks (top left sextet), causing transient loss of photoreceptor outer segments (bottom left), as well as severe visual field loss (top right). After treatment, the photoreceptors are partially reconstituted (bottom right)

Choriocapillaris inflammatory non-perfusion bears consequences on the outer retina for which it is the principal supply of oxygen and nutrients. While the RPE is relatively resistant to these choriocapillary perfusion disturbances, the external segments of the photoreceptors are much more fragile as a result of these metabolic changes and are easily damaged. SD-OCT morphologically shows the loss of the outer segments which co-localises and corresponds to choriocapillaris non-perfusion on ICGA. Presently, detection of these findings is still mostly limited to the posterior pole with the instruments available for common clinical practice. The advantage of this methodology is that it is a non-invasive tool that can be repeated easily and is useful for the follow-up of lesions (Fig. 11).

The importance of SD-OCT can be appreciated for secondary choriocapillaritis entities such as acute syphilitic posterior placoid chorioretinopathy measuring its effect on the outer retina [15]. Patients typically show outer retinal abnormalities, such as nodular thickening of the RPE with loss of the linear outer segment/RPE junction, disruption of the inner segment/ outer segment band, as well as loss of the external limiting membrane, accumulation of subretinal fluid ad punctate hyperreflectivity in the choroid. The SD-OCT findings are characteristic and may lead to the correct diagnosis without further unnecessary tests [16].

#### Enhanced depth imaging OCT (EDI-OCT)

**Fig. 12:**
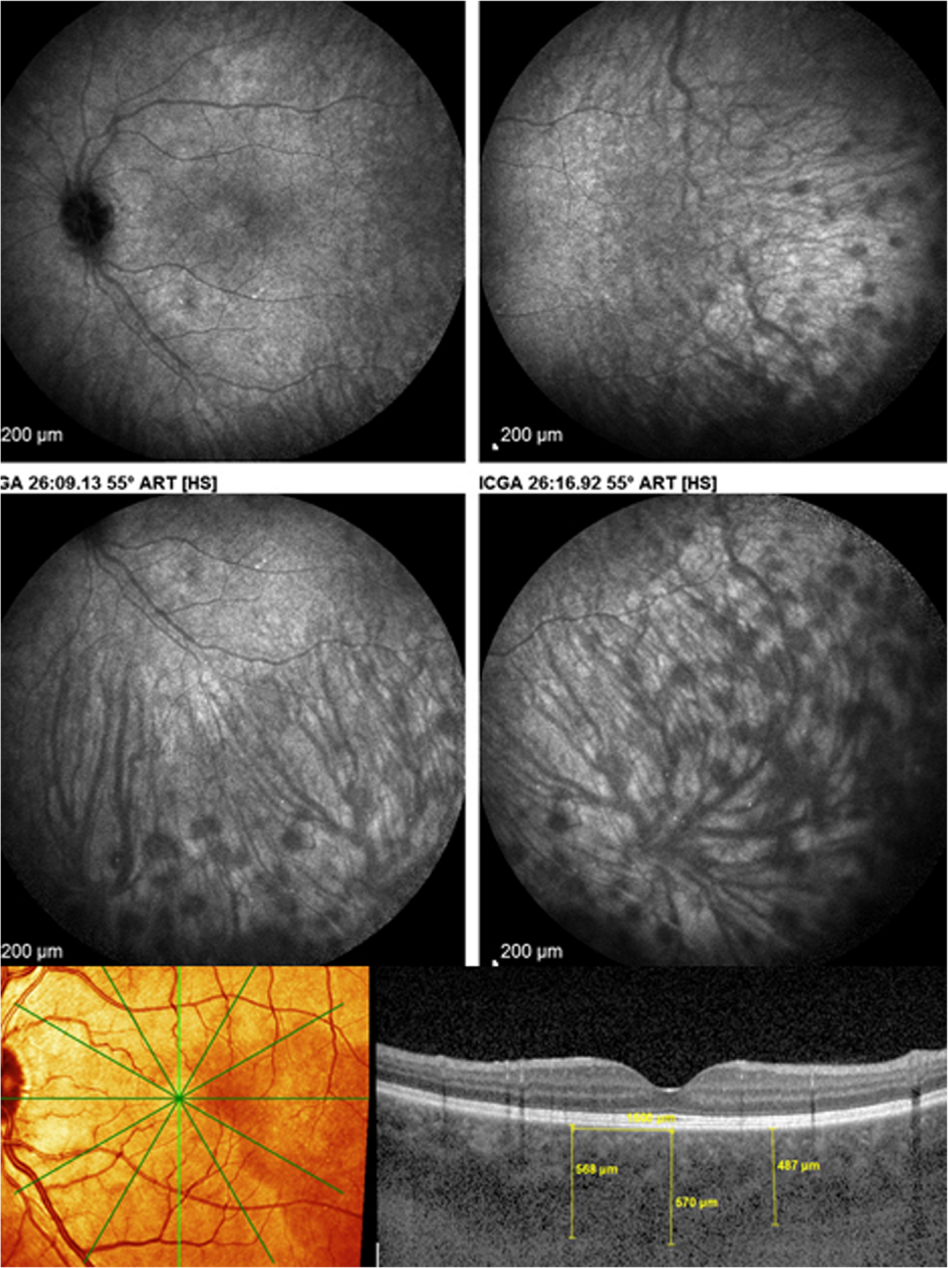
EDI measurement of choroidal thickness in a case of VKH disease. Numerous HDDs are shown in the top quartet of frames, that cause choroidal thickening which can be measured (bottom picture)

The technique of EDI-OCT was first described in 2008 and allows to measure choroidal thickness [17]. Its main use in choroiditis concerns principally stromal choroiditis. The choroid is thickened in acute stromal choroiditis including VKH disease, Sympathetic Ophthalmia and HLA-A29 BRC (Fig. 12). It is complementary to ICGA but gives information limited to the posterior pole with the advantage of being non-invasive. In chronic disease EDI-OCT shows thinned choroids and is less reliable than ICGA to show recurrent lesions. Like ICGA, EDI-OCT is very useful for early diagnosis and to monitor efficacy of therapy in stromal choroiditis but for this purpose it is also less sensitive than ICGA [18–20]. However, since ICGA is bi-dimensional, SS-OCT can provide more accurate images with precise measurement of depth and morphology of the stromal choroidal lesions.

#### OCT angiography (OCT-A)

**Fig. 13:**
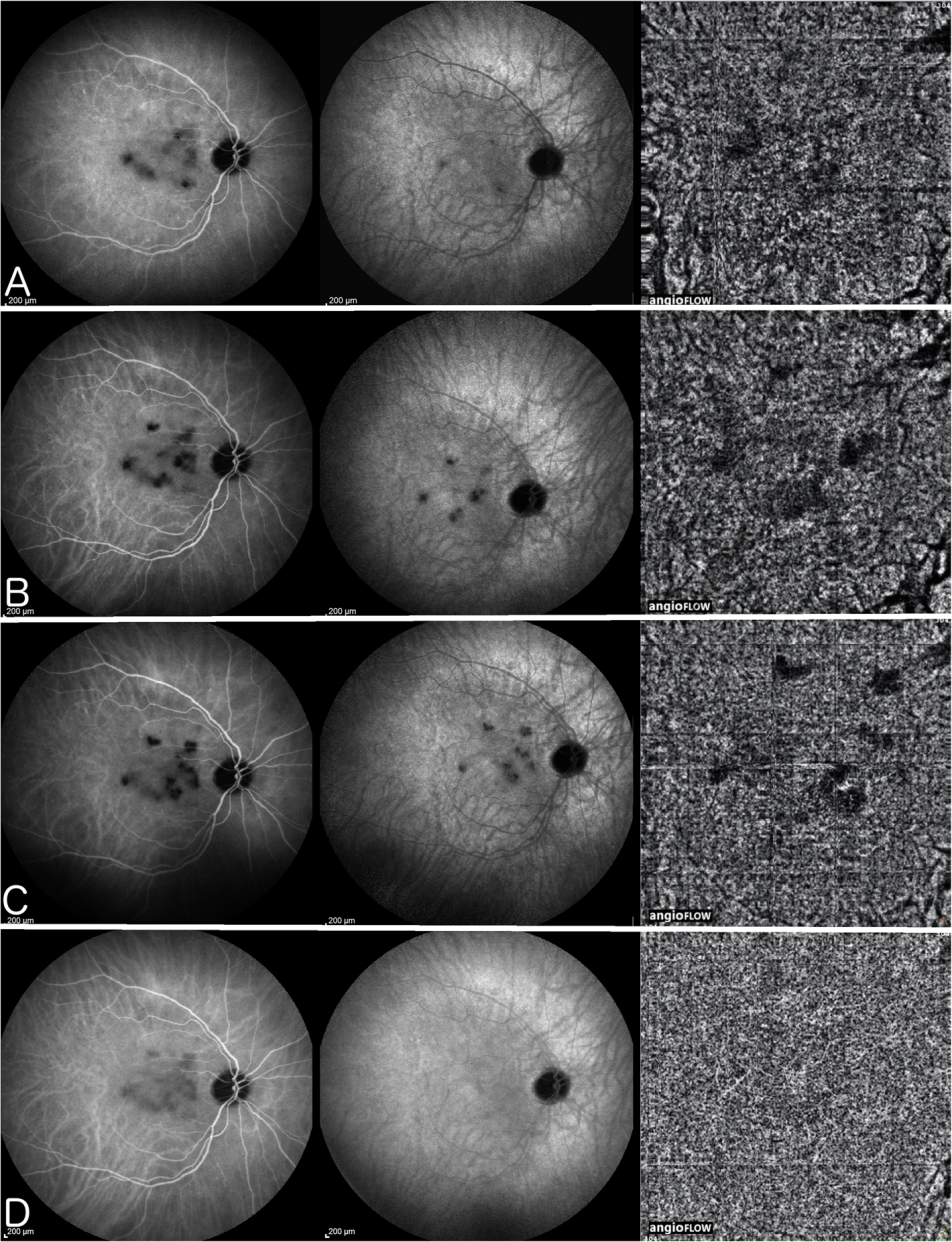
Serpiginous choroiditis - Parallel images of intermediate phase ICGA (left column), late phase ICGA (middle column) and 6x6mm OCT-Angiography (right column) of the right eye during follow-up ICGA of the right eye reveals macular hypofluorescent lesions better seen in the intermediate phase ICGA (left column) corresponding to choroidal hypo or non-perfused areas. These lesions increase in size and number (A) (B) (C) until aggressive immunosuppression is introduced (D); OCT-A pictures evolve in parallel with ICGA frames with faint dark areas visible (A, B, and C – right column), and disappearing after aggressive immunosuppression is introduced (D, right); involvement of extended areas depending on larger vessels, probably precapillary arterioles, are needed to produce a drop-out on OCT-A.

OCT-A is based on recording flow in vessels which is the principle that is generating imaging of retinal or choroidal vasculature without dye injection. Vessels in which flow is low cannot be detected by OCT-A. Consequently, it can only detect vasculature with sufficient flow. The vaso-dynamic behaviour of the end-capillary net of the choriocapillaris is characterised by very low flow and low resistance and can therefore not be detected by OCT-A. Vessel drop-out can be recorded by OCT-A in choriocapillaritis as long as it concerns vessels with sufficient flow, which is the case for choriocapillaritis entities such as APMPPE, MFC and SC where pre-capillary arterioles and/or larger vessels are involved. Indeed, Deutman, early in the discovery of the choriocapillaritis diseases, had understood that it was the obstruction of the pre-capillary arterioles that were at the origin of non-perfused choriocapillaris lobules in APMPPE, which he more appropriately called AMIC (acute multifocal ischaemic choriocapillaropathy) [14]. We showed that vessels with a sufficient size have to be involved to detect circulation drop-out such as is the case for SC. Even in this situation the non-perfused area was ill-defined on OCT-A frames and better identified by ICGA (Fig. 13) [21].

The end-capillary network has to be considered as a sponge slowly impregnated and emptying into the post-capillary venules in a low flow, low resistance hemodynamic fashion not detected by OCT-A. Therefore OCT-A is ill-suited to image this part of the choriocapillaris complex and absence of drop-out cannot be interpreted as an intact end-capillary network as was recently hypothesised for MEWDS. This type of very distal non-perfusion can however be detected using intravascular ICG dye by ICGA. The SS-OCT technology might shed some new light on the matter and will be the object of a separate article in this special issue on non-infectious choroiditis.

#### Blue light fundus autofluorescence (BL-FAF)

**Fig. 14:**
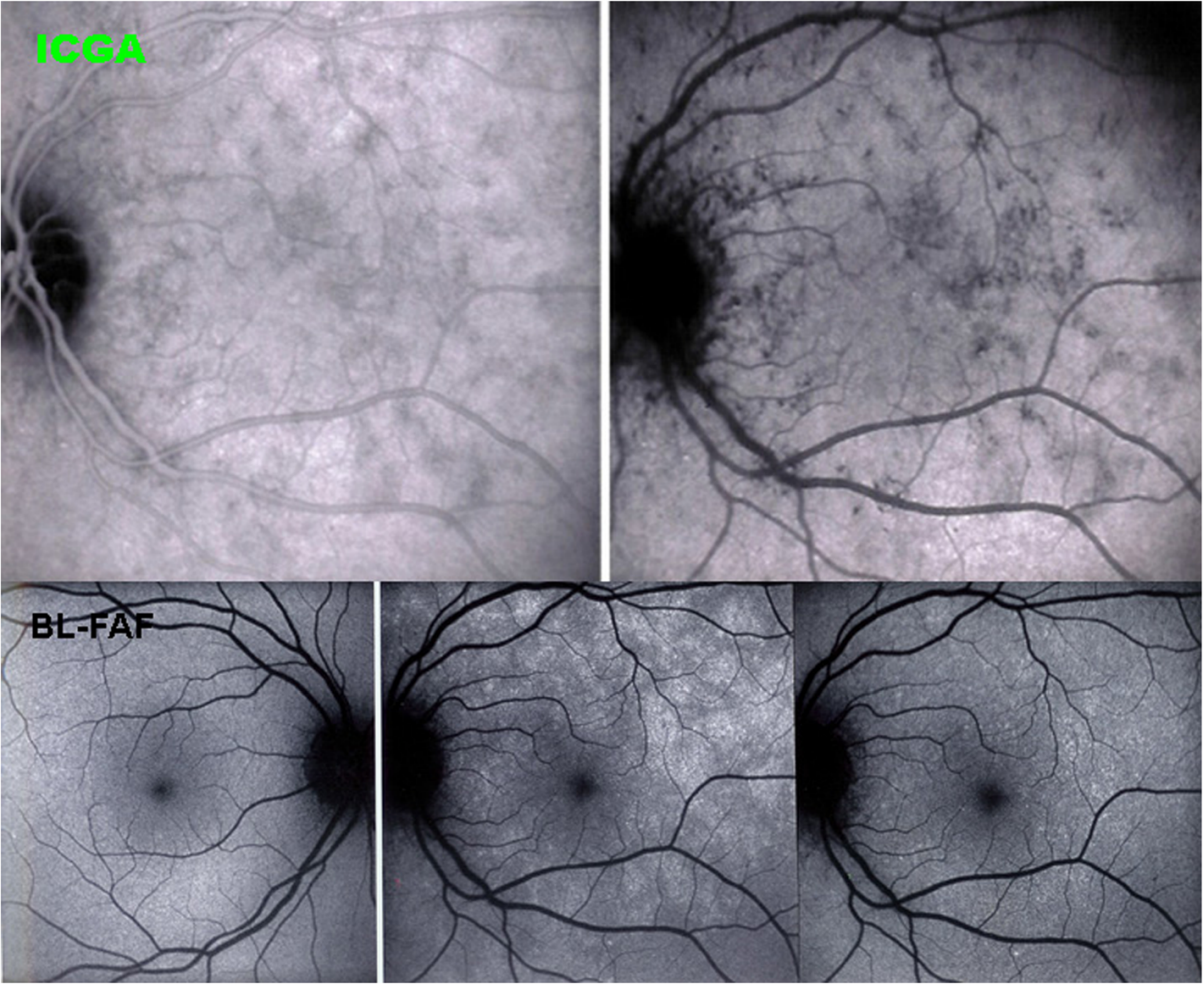
BL-FAF in a case of MEWDS. ICGA (two top frames) shows choriocapillaris non-perfusion. Normal BL-FAF in the healthy right eye (bottom left); Numerous hyperautofluorescent areas in the affected eye (bottom middle) which regress during convalescence (bottom right)

BL-FAF is classically performed to account for the effect of inflammatory choriocapillaris non-perfusion on the outer retina. Damage or loss of the photoreceptor outer segments due to ischaemia causes bleaching, unveiling the lipofuscin autofluorescence present in the RPE cells [22] (Fig. 14). Areas of fundus hyperautofluorescence have the same disposition as the hypofluorescent areas on ICGA, as the latter delineate choriocapillaris non-perfusion. Another imaging modality showing morphologically the damage or loss of photoreceptor outer segments in the affected areas is SD-OCT. The advantage of BL-FAF is that it delineates involved areas with similar if not better precision than ICGA and, additionally, is a non-invasive method [23].

## Glimpses of choriocapillaritis entities

**Fig. 15:**
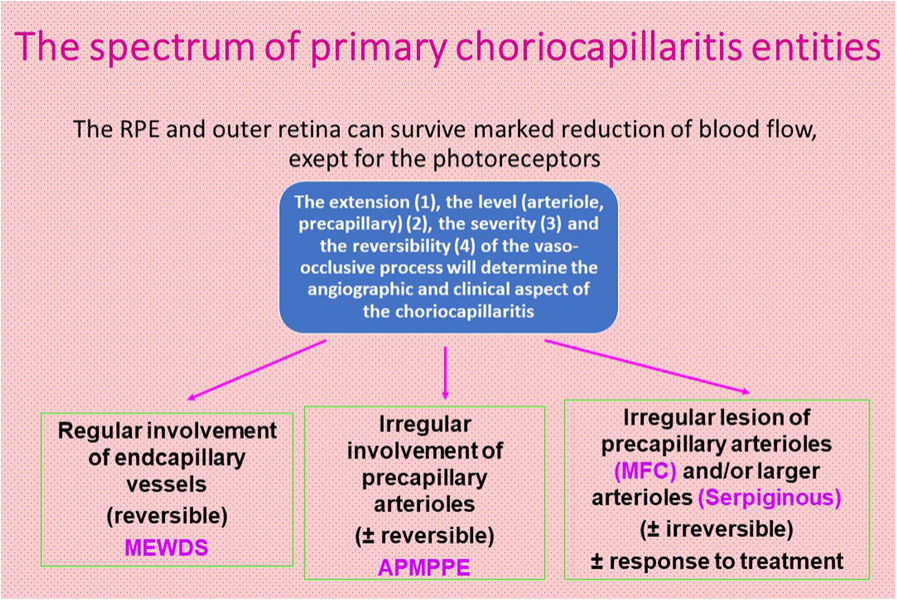
Schematic classification of PICCPs according to suspected location of vaso-occlusive event

Choriocapillaritis entities are classically subdivided into primary choriocapillaritis, also called primary inflammatory choriocapillaropathies (PICCPs), [11] including conditions for which the trigger is not known and supposed to be of viral origin, as many of these cases report flu-like symptoms preceding the choriocapillaritis. The most well-known conditions in this group are Multiple Evanescent White Dot Syndrome (MEWDS), Acute Posterior Multifocal Placoid Pigment Epitheliopathy (APMPPE) or Acute Multifocal Ischaemic Choriocapillaritis (AMIC), Idiopathic Multifocal Choroiditis (MFC) and Serpiginous Choroiditis (SC) (Fig. 15). In the group of secondary inflammatory choriocapillaropathies the trigger for the development of choriocapillaritis is known such as in Acute Syphilitic Posterior Placoid Chorioretinitis (ASPPC) or in tuberculosis-related serpiginous choroiditis.

### Primary choriocapillaritis or PICCPs

PICCPs are characterised by various phenotypes probably determined by the pattern of choroidal perfusion disturbance, from larger pre-choriocapillaris vessels at the origin of more severe forms such as MFC and SC, to end-choriocapillary vessels producing less severe forms such as MEWDS. These patterns also determine the evolution of the different entities, spontaneously reversible in case of MEWDS, and irreversible in case of MFC and SC, unless quick inflammation suppressive treatment is introduced. APMPPE/AMIC is situated between these two extremes depending on the severity of involvement that can be quite different from one case to the other. Moreover, some cases do not correspond to one of the 4 classically described entities, MEWDS, APMPPE/AMIC, MFC and SC and have therefore to be followed closely, as their phenotype cannot predict their evolution in a similar fashion as the well-determined entities.

This overview is not meant to give exhaustive information on the different non-infectious choroiditis diseases but to highlight their specificities and classify them according to disease mechanisms.

#### MEWDS

MEWDS, first described by Jampol and Sieving and Coll in 1984 [24, 25] is at the benign end of the choriocapillaritis spectrum probably because the level of the vascular occlusion process involves the most distal part of the choriocapillaris complex and does not involve widespread areas but multiple circumscribed non-confluent areas [26]. This explains both the often-limited damage and the reversibility of the disease, although this can lead to complications such as choroidal neovascularization (CNV) [27]. Nevertheless, it causes (limited) damage to the photoreceptors, as shown both by SD-OCT and BL-FAF. As indicated earlier, there is practically no flow in this distal sponge-like structure which cannot be imaged by OCT-A. This fact was at the origin of the interpretation that MEWDS is not a choriocapillaritis despite the presence of ICGA hypofluorescence present in all patients of this study having been investigated by ICGA [28]. However, a circulating dye like ICG is able to demonstrate absence of filling in non-perfused areas, clearly showing that choriocapillaris is at the origin of the photoreceptor damage. In the convalescent stage of the disease these areas are again filled with ICG.

**Fig. 16:**
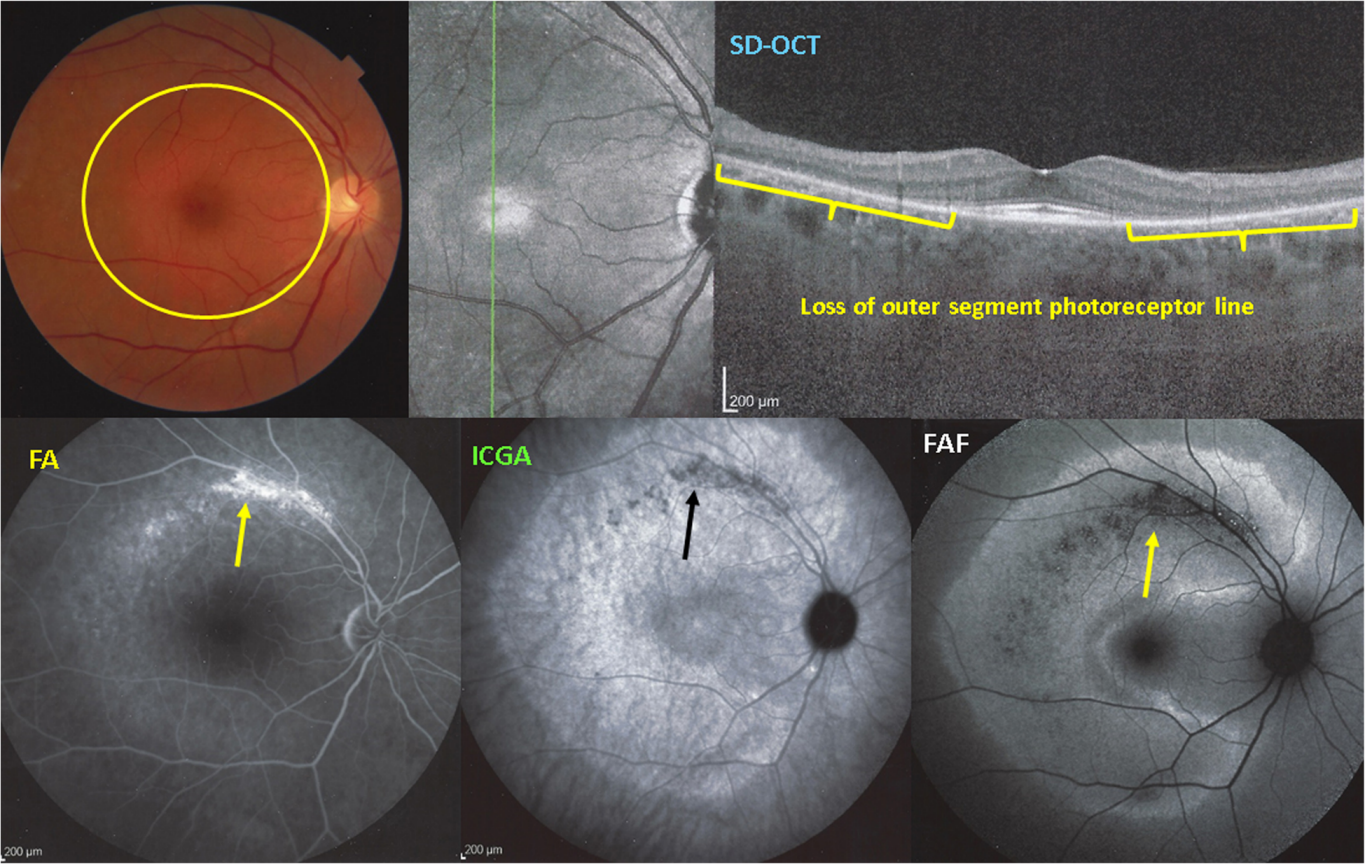
Example of AZOOR, a true primary phoreceptoritis with Imaging illustration of its clinicopathology. Fundus shows a pale discoloured halo around the fovea which retains a normal colour (top left, yellow circle) due to loss of photoreceptor photopigment. FA (bottom left picture) shows the same halo of discreet hyperfluorescence due to photopigment loss and an area of bright hyperfluorescence (window effect) along superior temporal arcade due to chorioretinal atrophy (dark on ICGA - bottom middle- and FAF – bottom right). ICGA (bottom middle) shows preserved choriocapillaris (except in the arciform area of chorioretinal atrophy) with increased fluorescence in the area of loss of the screen of photopigments which explains also fundus hyperautofluorescence (bottom right). SD-OCT (top right) shows the loss photoreceptor outer segments

Consequently, MEWDS cannot be a primary photoreceptoritis, which, in contrast, is the case of AZOOR [29]. Multimodal imaging allowed to demonstrate that AZOOR is a true primary disease of the external retina. As shown in Fig. 16, there is loss of outer segments of the photoreceptors with conserved choriocapillaris circulation as shown by conserved ICGA fluorescence except in the areas where there is complete chorioretinal atrophy.

As indicated here above, the hypothesis of a primary insult to the photoreceptors in MEWDS is still put forward by some centres. Nevertheless, additional sound evidence is needed.

#### APMPPE

APMPPE is a bilateral choriocapillaritis first described by Gass in 1968 who attributed the disease to the RPE [30].

In 1972, 4 years after the publication by Gass [31], and again in 1977 [32] and in 1983 [14], without the help of ICGA and based only on fluorescein angiographic signs, Deutman had understood the role of the choriocapillaris in PICCPs falsely attributed to the RPE as the primary structure involved [14]. For those who had the privilege to attend congresses where both Deutman and Gass were present, they were the spectators of the confrontation of these two theories and these two remarkable clinicians. Deutman had also understood that larger vessels had to be involved (precapillary arterioles) in the clinicopathology of APMPPE. Indeed, we know now that for the more severe choriocapillaritis entities such as APMPPE, MFC and SC the vaso-occlusive mechanism occurs at the precapillary level including larger areas of non-perfusion whereas in MEWDS the vaso-occlusive event takes place at a more distal level. As for MEWDS, there are differences of severity in APMPPE and to be safe systemic corticosteroids are not disadvised. Indeed, as the areas involved can be extended, chorioretinal scars can result in case of severe and prolonged ischaemia.

#### MFC

**Fig. 17:**
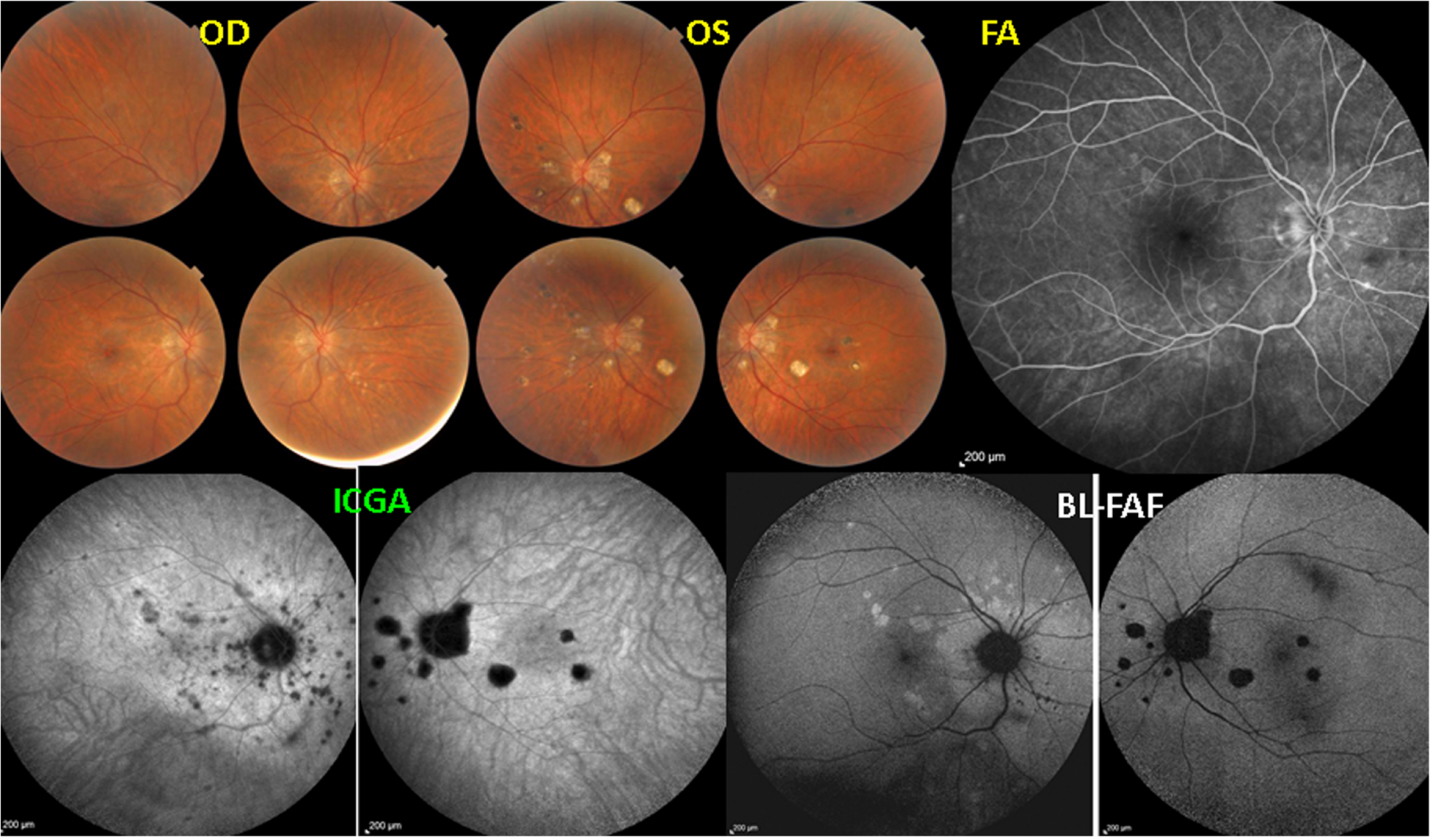
Multifocal Choroiditis. This patient had several episodes of MFC in the left eye with numerous remaining chorioretinal scars (fundus photograph OS, top middle, ICGA – OS, BL-FAF -OS. She consulted again for photopsias OD. No lesions visible on fundus OD, nor on FA, but numerous occult lesions visible on ICGA and BL-FAF OD. (1st and 3rd frame from the left, bottom)

Multifocal choroiditis (MFC) has been redefined in a nomenclature consensus editorial which regrouped different clinical entities with similar expressions that had been described separately in the past [33]. Such a systematisation was overdue. Indeed, when looking at the characteristics given in a review article for punctate inner choroidopathy (PIC) and MFC the two diseases could not be distinguished from each other [34]. The term of idiopathic multifocal choroiditis was proposed, including punctate inner choroidopathy (PIC) and other diversely described sub-entities such as multifocal inner choroiditis, recurrent multifocal choroiditis and others [33]. Furthermore, cases of “pseudo or presumed ocular histoplasmosis syndrome (POHS)” in patients with no serologic nor positive skin hypersensitivity test evidence for histoplasmosis should be considered as non-entities corresponding to MFC [33]. The characteristics of all the subtypes of MFC are the numerous small randomly distributed chorio-retinal scars and the recurrent behaviour of the disease as well as the high proportion of cases that develop secondary neovascular membranes which is much more frequent than in other choriocapillaritis entities. Multifocal choroiditis occurs in the same age groups as other choriocapillaropathies, namely in young to middle aged adults with myopic women being predominantly affected [35]. The disease can be unilateral or bilateral with recurrences over an extended period of time. Often, when seen for the first time because of photopsias, patients already present chorioretinal scars from previous unnoticed episodes. For such cases ICGA is crucial as it is the only precise and global imaging method to detect new subclinical lesions. BL-FAF is also helpful to localise and follow lesions (Fig. 17). MFC clearly belongs to the high-severity spectrum of PICCs, as lesions irretrievably lead to atrophic chorioretinal lesions. Immunosuppressive therapy with multiple agents is therefore recommended.

As in other choriocapillaris entities wide-field SS-OCT angio-analysis might contribute to the completion of the assessment of MFC and represent a further tool to refine its appraisal.

#### SC

SC is the most severe form of choriocapillaritis, probably because the vaso-occlusive involvement occurs in the more proximal larger arterioles producing extensive chorioretinal scarring [36]. Very aggressive immunosuppressive therapy is recommended often needing triple agent immunosuppression [37]. Before applying robust immunosuppression it is crucial to exclude proof of contact with *Mycobacterium tuberculosis* by performing an interferon-gamma release assay (IGRA). In case of positivity, the disease is an immunological choriocapillaritis induced by *Mycobacterium tuberculosis* also called serpiginoïd choroiditis [38], and vigorous anti-tuberculous antibiotics should be given in addition to aggressive immunosuppression.

#### Hybrid forms and PICCPs difficult to classify

**Fig. 18:**
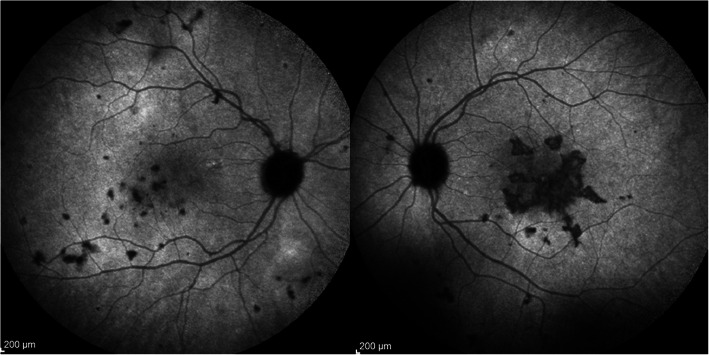
Case of mixed choriocapillaritis**.** The right eye shows the typical features of MFC, while lesions in the left eye take the aspect of SC

Not all PICCPs can be ranged into one of the well-determined entities. Hybrid forms can be seen that comprise signs corresponding to more than one form of disease (Fig. 18), which is the case of Amppiginous choroiditis [39]. In relentless placoid chorioretinitis, patients have the phenotype of APMPPE/AMIC but the evolution of serpiginous choroiditis [40]. Moreover, some cases are atypical and cannot be classified. They however present clinical and ICGA signs that clearly indicate choriocapillaritis. Careful monitoring by multimodal imaging is strongly recommended in order to intervene with corticosteroid/immunosuppressive treatment in case of deterioration.

#### Occurrence of different PICCPs in the history of the same patient

Rarely, different PICCPs can occur in the history of a patient constituting the so-called overlapping syndromes. MEWDS and MFC have been described in the same patient [41–43]. Such an occurrence tends to support the idea that these diseases belong to a common group with a clinicopathology due to circulatory disturbance of choriocapillaris and pre-choriocapillaris vessels. Blind spot enlargement has been described in all PICCPs probably due to peripapillary choriocapillaris non-perfusion [34, 44, 45]. This represents an additional element linking the choriocapillaris entities.

The different phenotypes of choriocapillaris may be explained by the level of choroidal circulatory impairment, its severity and its reversibility as shown on Fig. 15**.** If large precapillary vessels are involved, choriocapillaris non-perfusion is more wide-spread and ischaemia is more severe as in SC, MFC and some cases of APMPPE/AMIC. If the more distal end-choriocapillary vessels are involved, as in MEWDS, choriocapillaris non-perfusion is causing less ischaemia and consequently less damage to the outer retina and is more easily reversible. As the end-capillary choriocapillaris flow is a low-flow vascular network, choriocapillaris perfusion cannot be detected by OCT-A as images rely on sufficient flow and, hence choriocapillaris drop-out cannot be analysed by OCT-A.

### Secondary inflammatory choriocapillaropathies

#### Acute syphilitic posterior Placoid Chorioretinitis (ASPPC)

ASPCR, first described by Gass in 1990 [46], is a particular immune mediated involvement of the choriocapillaris caused by treponema pallidum, the agent of syphilis.

OCT shows quite characteristic findings that, together with ICGA, render further investigations unnecessary [16] (Fig. 11). It was shown that this entity can be halted by systemic corticosteroids without antibiotic therapy [47], questioning that the mechanism is purely infective but has indeed an immunological contribution [15]. Of course specific antibiotic anti-syphilitic therapy is the treatment that has to be applied which suppresses the syphilitic infection, the trigger for the choriocapillaritis.

#### Tuberculosis related serpiginous choroiditis (serpiginous-like choroiditis, serpiginoïd choroiditis, multifocal serpiginous choroiditis)

A similar mechanism is causing serpiginoïd choroiditis or Tuberculosis induced SC [38]. The particularity of this condition is that it has to be treated concomitantly with both multiple anti-tuberculous and multiple immunosuppressive agents.

## Glimpses of stromal choroiditis entities

The lesion process at the base of stromal choroiditis entities is the choroidal focus (granuloma) which impairs diffusion of the ICG dye and is seen in a negative, dark, hypofluorescent fashion which can be assimilated to an iceberg as, similarly, most of its space-occupying lesions are occult and not visible by classical investigational techniques except for ICGA or EDI-OCT (Fig. 8).

### Primary stromal choroiditis (PSC)

PSC entities are stromal choroidal diseases in which the inflammatory process is generated by the choroidal structures themselves, in opposition to secondary stromal choroiditis where the choroid is the chance localisation, so to say an innocent by-stander, of a systemic inflammatory disease such as sarcoidosis. In VKH disease (and SO) the inflammation originates exclusively from the choroid, produces first choroiditis, and then spills over to other structures such as the retina where exudative retinal detachments develop. The process results from an autoimmune reaction against melanin-associated antigens. In HLA-A29 birdshot retinochoroiditis (BRC), choroiditis also results from inflammation generated exclusively from choroidal structures involving the choroidal melanocytic islets. The triggering factor in BRC is unknown to date. The particularity of BRC is the concomitant although independent associated retinal vasculitis.

#### Vogt-Koyanagi-Harada disease (VKH)

VKH disease belongs to the pure primary stromal choroiditis entities, inasmuch as the unique source of inflammation is the choroidal stroma resulting from an autoimmune reaction against melanin-associated proteins. Numerous round regular HDDs relatively evenly distributed over the fundus seen on ICGA are the hallmark of the choroidal involvement in VKH disease (Fig. 8). In acute cases inflammation spills over into the retina producing exudative retinal detachments.

Until recently the importance to distinguish between initial-onset disease and chronic disease (due to delayed or insufficient treatment of initial-onset disease) was not sufficiently taken into account and this hampered the establishment of clear diagnostic criteria. The two sub-entities, initial-onset disease and chronic disease have to be considered separately as their course and response to therapy is different [48]. Recently practical diagnostic criteria for initial-onset VKH disease have been published [49] (Table 4) allowing for rapid diagnosis that, together with first-line steroidal and non-steroidal immunosuppression, is crucial to manage VKH disease and potentially obtain the cure of the disease [50].

**Table 4:** Diagnostic criteria for initial onset VKH Disease

1. No ocular trauma or surgery preceding onset of disease*	
2. Bilateral involvement (verified with ICGA and/or EDI-OCT) *	
3. Exclusion of other infectious, inflammatory or masquerading entities, in particular other stromal choroiditis entities (i.e. tuberculosis, sarcoidosis or syphilis) *	
4. Diffuse choroiditis evidenced by ICGA and/or EDI-OCT *	
5. Signs and symptoms of less than 4 weeks’ duration*	
6. Absence of clinical findings compatible with chronic disease (i.e. sunset glow fundus or integumentary signs (vitiligo, alopecia & poliosis) *	
**7. Exudative retinal detachments (evidenced by pooling and pinpoint leaking points on FA and ICGA) (very helpful criterion when present)**	
**8. Disc hyperfluorescence (helpful criterion)**	
**9. Neurological / auditory findings (meningismus, tinnitus, acute hearing loss) (helpful criterion)**	

*Essential criteria

#### Sympathetic Ophthalmia (SO)

SO results from the same immunopathological mechanism as VKH disease, namely an autoimmune reaction against antigens associated with melanocytes of the choroidal stroma. The only difference is that the autoimmune reaction has been triggered by a perforating eye injury or multiple operations.

#### HLA-A29 birdshot Retinochoroiditis (BRC)

**Fig. 19:**
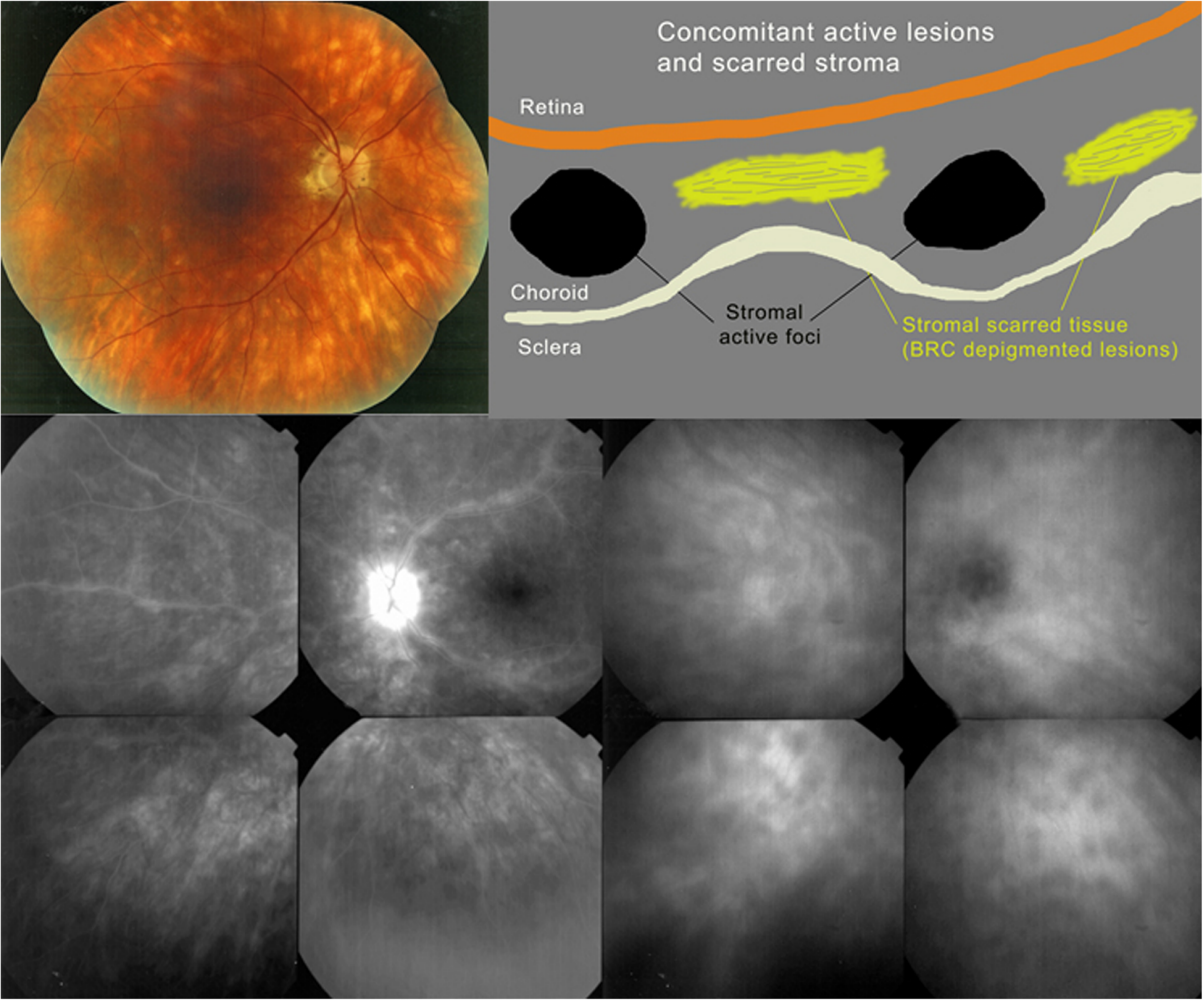
HLA-A-29 BRC. The typical fundal cream coloured rice shaped lesions are shown on the fundus picture (top left). The marked retinal vasculitis is shown on FA in the left bottom quartet. The choroidal foci are shown on ICGA in the right bottom quartet. The cartoon (inspired by Joan Mirò) (top right) explains that the cream-coloured lesions (yellow) do not correspond to the ICGA HDDs and do not appear on ICGA. HDDs correspond to the black dots on the cartoon

HLA-A29 birdshot retinochoroiditis was first described in 1980 by Ryan and Maumenee [51]. Significant progress in the understanding of the disease was achieved since the more than 40 years that elapsed since its first description [52]. What was lacking, as was the case for VKH, were clear diagnostic criteria, allowing to make early diagnosis with a high degree of confidence, which was recently proposed by a group of European BRC specialists [53] (Table 5). As for VKH, early and sustained therapy is needed in a great proportion of BRC cases [52]. The particularity of BRC is that in addition to primary stromal lesions there is concomitant retinal vasculitis a morbidity more difficult to treat than the choroiditis (Fig. 19). In recent years it has also been shown that thanks to new precise diagnostic criteria, especially thanks to ICGA, BRC can be diagnosed and treated before “birdshot depigmented rice-shaped lesions” are present and that these lesions can be prevented from appearing altogether by applying appropriate and prolonged treatment [54].

**Table 5:** European based simplified diagnostic criteria for BRC*

**1. Presence of vitritis in one or both eyes (required)**	
**2. Presence of retinal vasculitis in one or both eyes (required)**	
**3. Stromal choroiditis, as evidenced by ICGA, in both eyes (required)**	
**4. HLA-A29 antigen positivity (required)**	
**5. Visual field anomalies in one or both eyes (supportive)**	
**6. Absence of extra-ocular inflammatory site (supportive)**	
**7. Presence of rice-shaped depigmented “birdshot lesions” (BRC fundus lesions) (strongly supportive but not required)**

### Secondary stromal choroiditis

Secondary stromal choroiditis has to be distinguished from PSC, as the disease mechanism is fundamentally different. In contrast to PSC, inflammation does not originate from the choroidal structures but is caused by a multi-organ systemic disease of inflammatory or infectious character such as sarcoidosis or tuberculosis.

The choroid is just the chance, random structure, the innocent bystander, where the systemic disease unfolds.

#### Sarcoidosis Chorioretinitis (SARC)

Ocular sarcoidosis is among the most frequently diagnosed uveitis entities. It can produce a retinitis or a choroiditis or, most frequently, a chorioretinitis. Although sarcoidosis is a multiorgan condition, the eye can be, at least apparently, the only involved structure. Therefore, diagnostic criteria applying to isolated ocular sarcoidosis have been decided by an international workshop gathering of experts from around the world, the international workshop for ocular sarcoidosis (IWOS) in 2006 in Tokyo [55, 56].

Multimodal imaging is able to detect the exact involvement of the ocular lesions along the course of the disease which can involve different structures during different episodes [57]. Precise dual FA/ICGA angiographic scoring has been shown to exactly determine the proportion of retinal and choroidal inflammatory involvement and allowed precise monitoring of the evolution of lesions [58]. In contrast to PSC, hypofluorescent dark dots are less evenly sized and are characterised by a more random distribution [59].

## Concluding comments – the end of the “white dot syndrome” era?

Thanks to pioneering pragmatism and to the availability of multimodal imaging, appraisal of non-infectious choroiditis has progressed substantially in the last 2–3 decades. It evolved from the purely phenomenological potpourri terminology of “white dot syndromes” solely based on similar fundus appearance, towards sub-division into specific groups based on similar clinicopathologies. The first imaging modality that allowed to understand disease mechanisms was ICGA at the origin of the distinction between choriocapillaritis and stromal choroiditis. Later new imaging modalities contributed to more precise understanding and monitoring of these conditions, including SD-OCT giving morphological information and EDI-OCT allowing to measure severity of stromal choroiditis. BL-FAF represented a useful complementary non-invasive imaging modality to detect and monitor diseased areas in choriocapillaritis and OCT-A contributed non-invasive haemodynamic information on retinal and choriocapillaris circulations. Hopefully, with the precise information gained thanks to these new imaging modalities, the unfortunate and useless term of “white dot syndromes” including entities that have nothing in common, will be abandoned in favour a more precise and specific classification.

## Abbreviations

FA: Fluorescein angiography
ICGA: Indocyanine green angiography
SD-OCT: spectral domain optical coherence tomography
OCT-A: OCT angiography
EDI-OCT: Enhanced depth imaging optical coherence tomography
RPE: Retinal pigment epithelium
HDDs: Hypofluorescent dark dots
PICCPs: Primary inflammatory choriocapillaropathies
MEWDS: Multiple Evanescent White Dot Syndrome
AMIC: Acute multifocal ischaemic choriocapillaropathy
APMPPE: Acute Posterior Multifocal Placoid Pigment Epitheliopathy
MFC: Idiopathic Multifocal Choroiditis
SC: Serpiginous Choroiditis
IGRA: Interferon-gamma release assay
ASPPC: Acute Syphilitic Posterior Placoid Chorioretinitis
BRC: HLA-A29 Birdshot Retinochoroiditis
PSC: Primary stromal choroiditis
VKH: Vogt-Koyanagi-Harada disease
SO: Sympathetic Ophthalmia
SARC: Sarcoidosis chorioretinitis
BL-FAF: Blue light fundus autofluorescence
CNV: Choroidal neovascularization
PIC: Punctate inner choroidopathy
POHS: Presumed ocular histoplasmosis syndrome
